# Nanocellulose–Graphene Derivative Composite Membranes: Recent Advances, Functional Mechanisms, and Water Purification Applications

**DOI:** 10.3390/membranes15120347

**Published:** 2025-11-21

**Authors:** Hui Zhang, Shuyuan Lin, Yating Pan, Xin Wang, Hanzhou Zhang, Shuhan Liu, Zhen Li, Ning Wei

**Affiliations:** School of Mechanical Engineering, Jiangnan University, Wuxi 214122, China; zhzjm1101@163.com (H.Z.); linsy723@163.com (S.L.); pytdyx1@163.com (Y.P.); wangxinchole06@163.com (X.W.);

**Keywords:** nanocellulose, graphene derivatives, composite membranes, interfacial mechanisms, water purification

## Abstract

Nanocellulose–graphene derivative (NC–GD) composite membranes have attracted increasing attention as sustainable separation materials with high specific surface area, mechanical strength, and controllable interfacial chemistry. This review contextualizes the development of NC–GD composite membranes within advanced membrane technologies and summarizes recent progress in their structural design, interfacial mechanisms, and water purification applications. The synthesis and assembly of nanocellulose and graphene derivatives are analyzed, focusing on how surface functionalization regulates interfacial compatibility and transport pathways. Comparative evaluation of fabrication approaches—including vacuum filtration, layer-by-layer assembly, and solution casting—highlights their influence on structural uniformity and permeability. Key findings indicate that hydrogen bonding, electrostatic coupling, and π–π interactions govern the layer stability of composite membranes and the synergistic formation of nanochannels (by NC and GDs), thereby enabling efficient water permeation, selective separation, and fouling resistance. Overall, NC–GD membranes exhibit outstanding performance in heavy metal adsorption, dye removal, oil–water separation, and antibacterial treatment, representing a promising platform for next-generation sustainable water purification systems.

## 1. Introduction

Water is fundamental to human society, driving economic development and sustaining ecosystems. However, global water scarcity has become a critical barrier to sustainable growth. According to the United Nations World Water Development Report (2024), approximately 3.6 billion people experience water shortages for at least one month each year, and this number is projected to reach 5 billion by 2050, accounting for nearly 60% of the global population [1]. The Food and Agriculture Organization of the United Nations report (2020) further reveals that per capita freshwater availability has declined by more than 20% over the past two decades, with about 1.2 billion people living in severely water-stressed agricultural regions [2]. The uneven distribution of water resources exacerbates extreme hydrological events (e.g., droughts and floods), while worsening water pollution further reduces freshwater availability, posing severe challenges to agriculture, industry, and human health [3].

Membrane separation has emerged as an essential technology for water purification owing to its high separation efficiency. Nevertheless, conventional polymeric membranes—such as polyether sulfone (PES) and polyamide (PA)—still face inherent limitations, including fouling accumulation and the inevitable trade-off between permeability and selectivity. Tomczak et al. [4] reported that polyvinylidene fluoride (PVDF) and PES ultrafiltration membranes suffered substantial fouling caused by oil deposition and corrosion byproducts during long-term wastewater treatment. Even when the oil concentration decreased from 160 mg L^−1^ to 100 mg L^−1^, the flux of PVDF membranes increased only slightly from 57 to 77 L·m^−2^·h^−1^, and irreversible performance degradation persisted after multiple chemical cleanings. In pig-manure digestate filtration, Yue et al. [5] observed that among four polymer membranes, polyacrylonitrile exhibited the best flux-retention capability, whereas PES showed the poorest performance. The flux recovery of PVDF membranes after sodium hypochlorite cleaning reached approximately 73.1%, while that of polyacrylonitrile was only 30.1%, highlighting significant material-dependent differences in fouling and cleaning responses. Zhang and Geise et al. [6] further identified a distinct inverse relationship between water flux and salt rejection in PA/nanofiltration/reverse osmosis (RO) membranes: an increase in water permeability is typically accompanied by reduced NaCl rejection. For instance, seawater RO membranes generally exhibit permeation coefficients of 2–3 L·m^−2^·h^−1^·bar^−1^ with high salt rejection, whereas brine RO membranes reach 3–5 L·m^−2^·h^−1^·bar^−1^ at the expense of lower selectivity [7]. These intrinsic trade-offs have motivated the development of nanomaterial-based composite membranes with improved performance and durability.

**Figure 1:**
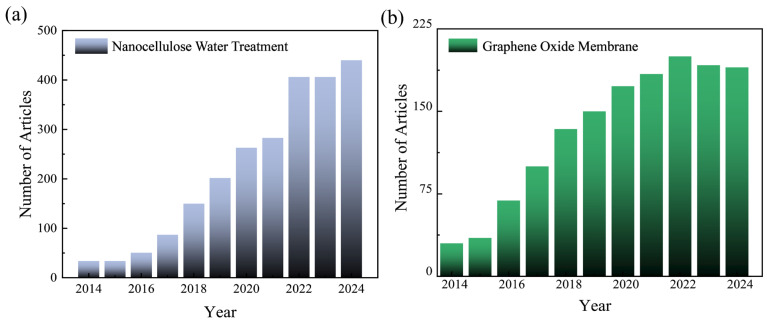
Annual trend of research papers on NC and GO for water treatment from 2014 to 2024. (**a**) Annual publication volume related to “nanocellulose water treatment”; (**b**) annual publication volume related to “graphene oxide membranes”.

Nanocellulose (NC) has emerged as a promising material in membrane science due to its renewable and sustainable origin, high specific surface area, abundant surface hydroxyl groups, excellent mechanical strength, and intrinsic hydrophilicity. It not only enhances membrane mechanical robustness and fouling resistance but also enables the effective regulation of pore size and water transport channels. Mautner et al. [8] reported that the pore structure of NC-based membranes largely determines their water flux, spanning from ultrafiltration to nanofiltration regimes, while hydroxyl functionalities contribute to superior hydrophilicity and anti-fouling behavior. Boonyaporn et al. [9] demonstrated that bacterial NC-derived membranes could achieve a high water flux of 0.70 ± 0.27 L·m^−2^·h^−1^·kPa^−1^ and a bovine serum albumin rejection of 98.0 ± 2% at a low pressure of ~3.4 kPa, highlighting their outstanding fouling resistance. Similarly, Wu et al. [10] incorporated cellulose nanocrystals (CNC) or modified cellulose nanofibrils (CNF) into PVDF membranes, which significantly improved the porosity, hydrophilicity, and mechanical strength while alleviating the conventional flux-selectivity trade-off. Graphene derivatives (GDs) have also shown remarkable potential in membrane separation due to their two-dimensional ultrathin structure, high chemical stability, tunable surface functionality, and confined transport effects. Pre-crosslinked graphene oxide (GO)–*p*-phenylenediamine nanofiltration membranes (~40 nm thick, ~400 cm^2^ area) achieved a 99% rejection of multiple dyes at a flux of ~42 L·m^−2^·h^−1^·bar^−1^—nearly an order of magnitude higher than their uncrosslinked counterparts—while maintaining excellent stability under both acidic and alkaline conditions [11]. Another ultrathin GO membrane (~12 nm thick) exhibited a water flux of ~1505 L·m^−2^·h^−1^·bar^−1^ and an Evans Blue removal efficiency of ~98.7%, demonstrating a significant enhancement in the permeability–selectivity balance [12]. As shown in Figure 1, research on both NC- and GO-based membranes has exhibited a continuous upward trend over the past decade, reflecting sustained academic interest in their complementary properties. This convergence further underscores the growing prominence of NC–GD composites as a promising direction in advanced membrane development.

Given this background, it is timely to comprehensively summarize the advances in NC–GD composite membranes. Although several reviews have focused on membranes based on individual nanomaterials, including a recent comprehensive review by Mokhena et al. on NC–GO composites [13], integrated analyses addressing preparation strategies, interfacial mechanisms, and application prospects from a synergistic materials perspective remain limited. This review aims to elucidate the complementary interactions and cooperative effects between NC–GDwithin composite membranes, clarify their roles in mass-transfer regulation, anti-fouling performance, and structural stabilization, and highlight representative case studies in seawater desalination, pollutant removal, and emerging separation technologies. Unlike previous reports that mainly emphasize performance metrics, this review adopts an integrative structure–mechanism–application framework to rationalize the evolution of membrane behavior. The discussion provides both theoretical insights and practical guidance for the rational design of high-performance sustainable water treatment membranes and the continued advancement of membrane science.

## 2. Material Fundamentals

### 2.1. Nanocellulose

**Figure 2:**
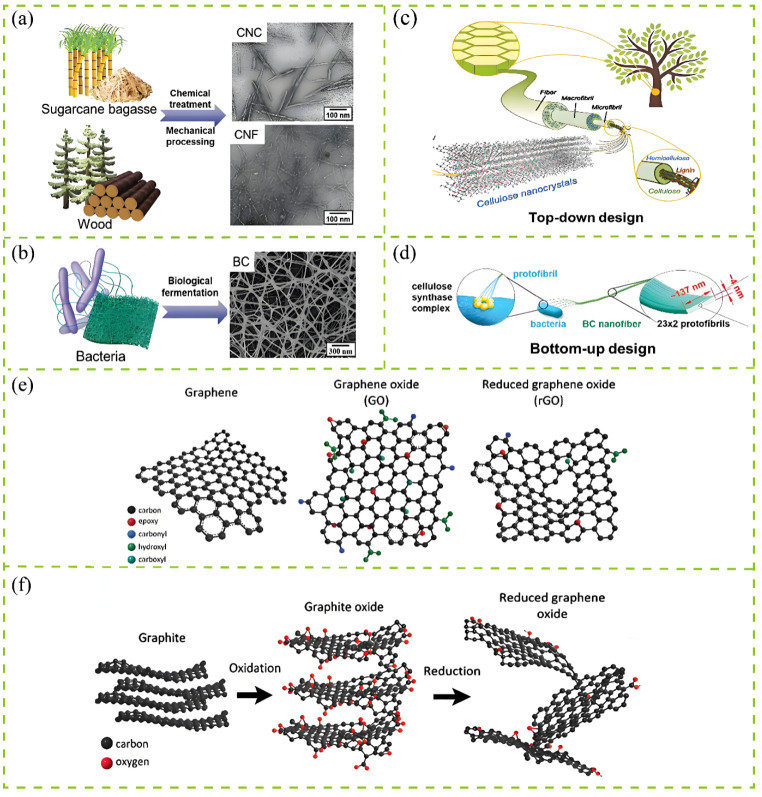
Material preparation process diagram. (**a**) Preparation routes from natural biomass; (**b**) bacterial cellulose biosynthesis; (**c**) top-down fabrication strategy; (**d**) bottom-up assembly strategy; (**e**) schematic diagram of chemical structures of graphene, graphene oxide, and reduced graphene oxide; (**f**) roadmap for preparation of GO and rGO from graphite. Images (**a**–**d**) reproduced with permission from Ref. [36]; images (**e**,**f**) reproduced with permission from Ref. [37].

NC is a nanoscale cellulose-derived material obtained from natural sources and is generally classified into three main types: CNC, CNF, and bacterial cellulose (BC) [14,15]. CNCs are typically obtained from plant fibers through chemical hydrolysis or oxidation, whereas cellulose nanofibrils CNFs are produced by mechanical or chemical disintegration of cellulose, as shown in Figure 2a. Both follow top-down routes with complex procedures and limited efficiency, corresponding to the fabrication strategy in Figure 2c. In contrast, bacterial BC is synthesized through a bottom-up biosynthetic pathway from glucose monomers, as illustrated in Figure 2b,d, yielding nanofibers up to ~100 μm in length and ~137 nm in width. Owing to its high specific surface area, remarkable mechanical properties, renewability, and versatile surface chemistry, NC has shown exceptional promise in membrane separation applications [16,17]. CNCs, with their uniform rod-like morphology and abundant hydroxyl functionalities, enhance membrane mechanical strength and selectivity. CNFs, composed of entangled nanoscale fibrils, form three-dimensional network structures that improve pore uniformity, permeability, and film-forming ability while increasing material flexibility [18]. BCs, synthesized in situ by microorganisms, possess high purity, crystallinity, and an interconnected 3D structure, yielding outstanding water flux and structural integrity [19,20]. Collectively, these three types of NC offer complementary advantages for reinforcing, structuring, and functionalizing membrane materials, thereby providing a versatile toolbox for optimizing membrane performance.

In recent years, a new generation of functionalized NC derivatives has emerged through molecular tailoring and hybridization strategies. Controlled esterification, etherification, oxidation, and click-chemistry reactions enable the introduction of carboxyl, sulfonate, phosphate, quaternary ammonium, and azide groups onto CNC, CNF, or BC surfaces, constructing a tunable functional NC platform with adjustable charge density, hydrogen-bonding capability, and steric configuration [21]. In parallel, in situ mineralization, coordination intercalation, and dynamic covalent crosslinking have been employed to anchor metal ions, organic ligands, or oligomers onto the NC backbone, forming NC–inorganic/organic hybrids that achieve rigid–flexible synergy and multi-scale pore tunability [22,23]. These emerging NC derivatives preserve the mechanical strength and renewability of native NC while introducing controllable surface charge, reversible stimuli response, and abundant active sites. They facilitate dual polarization–affinity gradients within membranes, tune pore–wall surface energy, and create confined water channels, offering expanded design dimensions for next-generation separation membranes emphasizing pore regulation, interfacial polarization, and functional integration.

The evolution of NC synthesis from single-step acid hydrolysis to diversified composite processes mirrors advances in green chemistry and material science. In the 1950s, Rånby first obtained uniform cellulose microcrystals, later termed CNCs, via sulfuric acid hydrolysis, establishing the foundation for modern NC research [24]. Subsequently, Marchessault et al. identified the optical birefringence of CNCs [25], and Revol et al. discovered their liquid–crystalline behavior in the 1990s [26], which expanded their use in functional materials. In the 1980s, Turbak and Herrick developed the microfibrillation method, in which cellulose pulp was disintegrated into submicron fiber networks using high-pressure homogenization [27,28]. This approach minimized chemical usage but required high energy input, paving the way for industrial-scale studies. After the 1990s, acid hydrolysis techniques became standardized. Beck-Candanedo and Gray systematically examined the effects of acid concentration, temperature, and reaction time on the CNC size, surface charge, and dispersibility, ultimately establishing sulfuric acid hydrolysis as the benchmark process [29]. Entering the 21st century, environmental and energy concerns shifted focus toward greener pretreatment and sustainable synthesis routes. Between 2006 and 2007, Isogai and co-workers introduced the 2,2,6,6-tetramethylpiperidin-1-yl-oxyl (TEMPO)-mediated oxidation method, which generates carboxylated cellulose by selectively oxidizing primary hydroxyl groups. This modification weakens inter-fibril hydrogen bonding, enabling efficient disintegration into CNFs under mild mechanical forces. The TEMPO process, characterized by a low energy demand and excellent colloidal stability, rapidly became the mainstream CNF preparation route [30,31]. Recently, the introduction of green solvents and bio-based strategies has further advanced sustainable NC production. Ionic liquids and deep eutectic solvents, known for their high cellulose solubility and recyclability, have been increasingly used for cellulose pretreatment and nanostructuring [32,33,34]. Moreover, enzymatic hydrolysis coupled with mild mechanical dispersion has demonstrated both environmental friendliness and low energy consumption. Compared with traditional acid hydrolysis, these processes emphasize recyclability and scalability, making them a focal point of current research and industrial translation [35].

### 2.2. Graphene Derivatives

GDs are a versatile class of two-dimensional carbon materials derived from graphene, known for their exceptional physicochemical properties and tunable surface functionalities [38]. Common GDs include GO, reduced graphene oxide (rGO), and amino-functionalized graphene [39,40]. As illustrated in Figure 2e, graphene consists of a single atomic layer of carbon atoms arranged in a honeycomb lattice. Oxidation introduces oxygen-containing groups (hydroxyl, epoxy, and carboxyl) on the basal planes and edges of GO, enhancing its hydrophilicity and dispersibility. Subsequent reduction via chemical, thermal, or photochemical routes partially restores the sp^2^-conjugated structure of GO to yield rGO, improving the electrical conductivity and hydrophobicity. Figure 2f schematically depicts the typical preparation sequence: graphite is first oxidized to graphite oxide, exfoliated into GO sheets, and optionally reduced to rGO. This transformation visualizes the structural and functional evolution from graphite to GO and rGO, forming the foundation for understanding property modulation and synthesis control.

Recent progress has led to a diverse range of advanced GDs, including graphene quantum dots, plasma-assisted super-reduced graphene, and self-healing GO–ionic liquid hybrids. For example, the GO@Fe_3_O_4_ quantum dot composite integrates graphene quantum dots for high surface area, GO for enhanced dispersion, and Fe_3_O_4_ for magnetic recoverability. It efficiently removes Cr(III) and organic dyes such as Rose Bengal via adsorption and visible-light photocatalysis, showing high efficiency, fast degradation, and excellent recyclability for water purification membranes [41]. The combination of super-reduced graphene with MXene generates conductive–hydrophilic heterochannels that impart both anti-fouling and capacitive regeneration capabilities, maintaining >95% water flux after 30 cycles at 500 ppm NaCl [42]. Moreover, dynamically grafted GO–ionic liquid systems exhibit self-repairing properties, enabling crack healing under mild conditions for potential applications in self-healing sensors and wearable electronics [43]. These innovations—from quantum-level constructs to macroscopic interfaces—have transformed graphene derivatives from simple interlayer materials into multifunctional platforms offering tunable charge, size, interfacial, and self-healing properties. GDs are thus becoming key structural units in the design of next-generation high-performance, intelligent, and sustainable separation membranes.

The synthesis of GDs has evolved from traditional oxidative methods to a variety of exfoliation and growth techniques. Early approaches such as Brodie’s (1859) and Staudenmaier’s (1898) methods relied on strong acids and chlorates for graphite oxidation, they but suffered from safety and reproducibility issues [44,45]. The Hummers method (1958), employing potassium permanganate and sulfuric acid, became the classic route for preparing graphite oxide [46]. Later modifications replaced nitrate with phosphoric acid and optimized temperature control, improving oxidation efficiency while minimizing toxic byproducts [47]. Beyond chemical oxidation, mechanical exfoliation produced the first single-layer graphene in 2004, though scalability remained limited [48]. Subsequently, chemical vapor deposition enabled the controlled growth of large-area graphene films on metallic substrates, suitable for electronic and separation applications [49]. Liquid-phase exfoliation using ultrasonication or high-shear dispersion of graphite in selected solvents emerged as a scalable route for producing GO and graphene dispersions [50]. In the past decade, electrochemical exfoliation and electrochemical oxidation techniques have attracted increasing interest, allowing controllable interlayer separation driven by electric fields and tunable oxidation levels [51]. Collectively, these strategies have evolved GD synthesis from single-step oxidation to a diverse suite of chemical, mechanical, vapor-phase, and electrochemical routes, establishing flexible process systems tailored to different application scenarios.

## 3. Preparation Methods for NC-GD Composite Membranes

Different types of NC and GDs exhibit distinct physical morphologies and surface chemistries, which profoundly influence their compatibility and structural evolution during membrane fabrication. For example, the rigid rod-like morphology of CNC promotes regular compact stacking during vacuum-assisted filtration, making them ideal for fabricating highly selective layered membranes. In contrast, the flexible and entangled nature of CNF, together with their excellent dispersibility, enables the formation of uniform interfacial networks in layer-by-layer (LbL) assembly processes. BC, with its three-dimensional interconnected pores derived from microbial biosynthesis, provides superior pore continuity and mechanical reinforcement when employed in solution casting or phase inversion methods.

The oxidation–reduction state of graphene derivatives also governs their processing behavior. GO, characterized by its abundant oxygen-containing groups and hydrophilicity, readily disperses in aqueous systems, making it highly suitable for vacuum filtration or self-assembly. Reduced rGO and aminated GO, being relatively hydrophobic, are more compatible with solvent-driven processes such as solution casting and phase inversion. Therefore, the subsequent sections analyze the suitability of various NC–GD combinations under different fabrication routes, elucidating the underlying mechanisms of structural regulation and process selection. These relationships are systematically compared in Table 1.

### 3.1. Vacuum-Assisted Filtration

**Figure 3:**
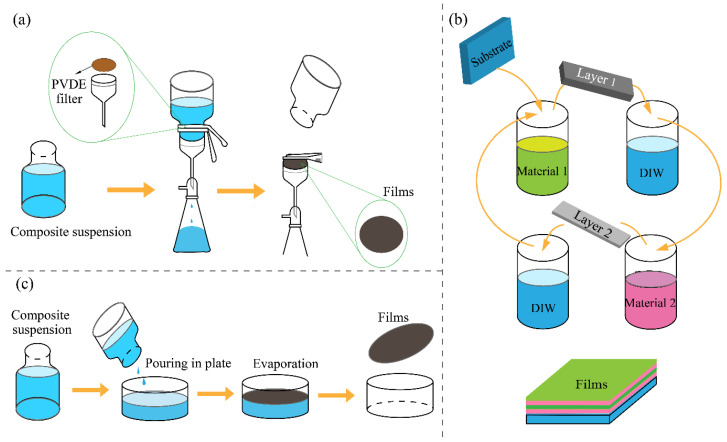
Preparation method of NC-GD composite membranes. (**a**) Vacuum-assisted filtration; (**b**) layer-by-layer self-assembly; (**c**) solution casting.

Vacuum-assisted filtration is among the most widely employed techniques for constructing NC–GD composite membranes, with a schematic of the process provided in Figure 3a. This process exploits the aqueous dispersibility of both components, allowing negative pressure to drive suspended particles onto a substrate, where they form compact and ordered multilayer structures. It is particularly well-suited for systems combining CNCs or CNFs with GO. Under vacuum, the rigid CNC rods assemble into a mechanically stable scaffold that prevents GO layers from undergoing excessive stacking or channel collapse, thereby maintaining ordered lamination and stable transport pathways.

When flexible CNF is used, ultrasonic pre-dispersion (typically 0.5–1 wt %) is recommended to avoid aggregation, and the filtration rate must be carefully controlled to balance membrane compactness and water flux. Due to its lower hydrophilicity, rGO is often co-blended with GO or subjected to mild oxidation to improve dispersion before filtration. Numerous studies have demonstrated the effectiveness of this method in tuning membrane architecture and performance. Chen et al. [55] fabricated rGO–CNC membranes through sequential vacuum filtration of CNC and GO dispersions followed by chemical reduction. The rigid CNC framework effectively supported the GO layers, yielding nanofiltration membranes with high selectivity and structural stability. Zhu et al. [56] prepared self-standing CNF–GO membranes using vacuum filtration followed by thermal treatment to adjust interlayer spacing, achieving efficient dye rejection and enhanced mechanical integrity. Other studies have incorporated interfacial modifiers such as chitosan to strengthen GO–CNF adhesion. For instance, CNF–GO/chitosan multilayer membranes exhibited improved water flux and fouling resistance [57], while Kang et al. [58] tuned membrane thickness to balance flux and rejection, with the flexible CNF network providing additional transport channels. Similarly, Abolhassani et al. [59] prepared chitosan–GO membranes via vacuum filtration, where hydrogen bonding and electrostatic interactions between amino and carboxyl groups stabilized interlayers and suppressed swelling, maintaining high filtration efficiency in wastewater treatment.

Overall, vacuum-assisted filtration is applicable to various NC types (CNC, CNF, or BC) and allows versatile structural tuning via interfacial modifiers such as chitosan or its derivatives. It offers a simple, controllable, and environmentally friendly approach for fabricating GO–NC composite membranes with balanced permeability, stability, and fouling resistance.

### 3.2. Layer-by-Layer Self-Assembly

LbL self-assembly is particularly advantageous for CNF and GO systems, owing to their tunable surface charge and strong interfacial interactions. The high flexibility and surface hydroxyl density of CNF enable it to form uniform and continuous layers during alternate deposition cycles. Meanwhile, negatively charged GO can electrostatically interact with cationized CNC or amino-functionalized GO to build stable multilayer architectures. CNC, being more rigid, serves as a structural reinforcement phase that maintains dimensional stability, while CNF contributes flexibility and interlayer connectivity, improving the overall film integrity.

The abundant hydroxyl groups and good dispersibility of NC facilitate strong interfacial bonding with GO, leading to durable and uniform assemblies. Wågberg et al. [60] highlighted the pivotal role of NC in charge-driven LbL assembly. Fang et al. [61] further demonstrated that introducing polydopamine as an interfacial binder significantly enhanced the mechanical strength and water treatment efficiency of GO–CNF membranes. Similarly, Lee et al. [62] fabricated GO-cationized CNC multilayer membranes with precisely controlled thickness through multiple deposition cycles, achieving efficient ion separation. Raghuwanshi et al. [63] reported that the co-assembly of GO nanosheets and CNC nanorods produced stable composite architectures, revealing the fundamental self-assembly mechanism.

LbL self-assembly, as schematically shown in Figure 3b, maximizes the synergistic interactions between NC and GO, enabling nanoscale precision in structural design and performance control. It thus offers a powerful strategy for developing functional membranes with tunable selectivity, high mechanical integrity, and controlled interfacial chemistry.

### 3.3. Solution Casting

Solution casting is another common technique for fabricating NC-GD composite membranes, and a schematic of this process is presented in Figure 3c. In this method, pretreated NC and GO (or other GDs) are dispersed in a polymer solution to form a homogeneous suspension. The mixture is then uniformly cast onto a flat substrate—such as glass or a Teflon^®^ plate—and the solvent is allowed to evaporate naturally or under controlled drying, yielding a uniform composite film.

This approach is particularly suitable for combinations involving CNF or BC with well-functionalized GDs such as rGO or aminated GO. The flexibility and good dispersibility of CNF facilitate the formation of a stable solution suspension, while the partial hydrophobicity of rGO improves interfacial adhesion and resistance to swelling after film formation. CNC, owing to its rigidity, can serve as an inorganic reinforcing phase, though its concentration should remain below 1 wt % to prevent phase separation. The gradual solvent evaporation process allows sufficient time for molecular rearrangement and crosslinking, resulting in dense uniform composite membranes. Valencia et al. [64] prepared CNF–GO layered membranes via solution casting, achieving two-to threefold higher water flux and significantly enhanced mechanical stability compared with CNF-only membranes. The composite membranes also exhibited improved adsorption and retention capacities for metal ions and dyes. Other studies have incorporated CNC–GO as hydrophilic and anti-fouling additives into PVDF matrices, yielding high-performance modified membranes via casting [65]. Similarly, the incorporation of GO–CNC hybrids into PVDF membranes has been shown to improve the hydrophilicity and anti-fouling characteristics [66].

Solution casting is favored for its operational simplicity, controllable composition, and compatibility with various polymer matrices, which makes it particularly suitable for mechanistic investigations. However, its limitations include challenges in controlling membrane thickness, fine-tuning pore morphology, and scaling-up to continuous industrial production.

In summary, each fabrication technique for NC–GD membranes prioritizes distinct structural attributes and material compatibilities. Vacuum-assisted filtration yields highly ordered and compact architectures, LbL assembly permits nanoscale control over layer composition and interfacial properties, and solution casting facilitates versatile composite formulation with polymer matrices. A comparative analysis of key performance metrics—including water flux, solute rejection, and cycling stability—for representative membranes is provided in Table 2. For ionic substances, CNC–GO membranes [67] achieve >95% antibiotic rejection and 99.1% divalent ion (Mg^2+^/Ca^2+^) rejection; TOCNF–nanoGO composites [56] show a Cu(II) adsorption capacity of 68.1 mg/g; CNC–rGO/PEO membranes [68] deliver 98.3% NaCl rejection with minimal flux decline over time. In organic macromolecule separation, CNF–GO membranes [64] reach >90% dye rejection with fivefold flux enhancement vs. pure CNF; BC membranes [9] achieve 98 ± 2% BSA rejection. For biological/microbial substances, BC–rGO membranes [69] remove >95% organic matter and bacteria. In oil-phase separation, BC–GO membranes [70] achieve 90% oil–water separation efficiency within 5 s. These examples reflect the adaptability of NC–GD membranes to diverse targets, underscoring structure–performance correlations tailored by material and fabrication strategies.

## 4. Functional Mechanisms of NC–GD Composite Membranes

### 4.1. Interfacial Interactions

Interfacial interactions among nanoscale constituents fundamentally dictate the macroscopic performance of composite membranes [71]. In NC–GD systems, constructing stable and efficient interfaces is essential for achieving synergistic functionality. The nature and strength of interfacial forces directly influence filler dispersion, stress transfer, and the structural integrity of the membrane [72]. Noncovalent interactions dominate in this hybrid system, with hydrogen bonding serving as the primary stabilizing force.

**Figure 4:**
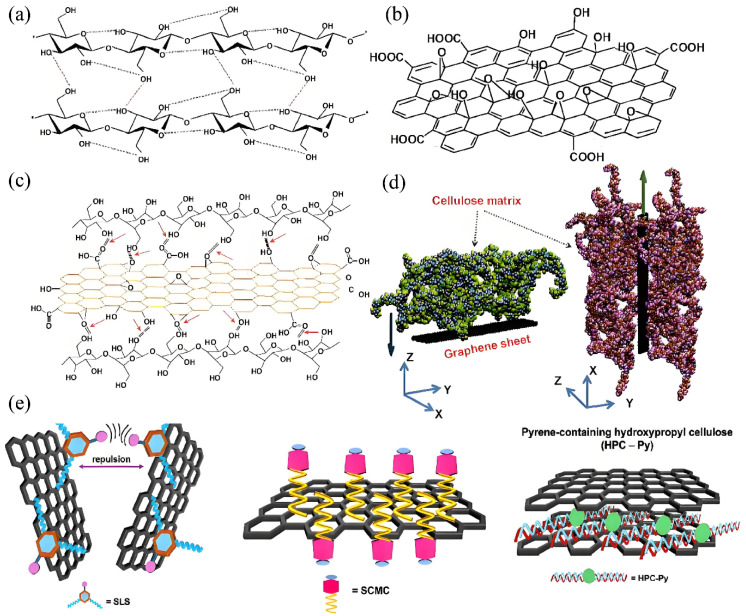
Interface mechanism diagram. (**a**) The intramolecular and intermolecular hydrogen bonding networks of NCs; (**b**) a possible structural model of graphene oxide; (**c**) schematic diagram of hydrogen bonding between regenerated graphene oxide nanosheets and NC molecular chains (indicated by red lines); (**d**) interface interaction model between graphene sheets and cellulose chain matrix; (**e**) non-covalent interaction diagram, based on chemical reduction of graphene, using sodium lignosulfonate, sodium carboxymethyl cellulose, and hydroxypropyl cellulose containing pyrene as non-covalent modifiers. Images (**a**–**d**) reproduced with permission from Ref. [84]; image (**e**) reproduced with permission from Ref. [85].

Dense intra- and intermolecular hydrogen-bond networks within NC chains (Figure 4a) provide intrinsic structural stability and establish the basis for subsequent interactions with graphene derivatives. NC chains are rich in hydroxyl groups, whereas graphene derivatives such as GO bear oxygen-containing functionalities—hydroxyl, epoxy, and carboxyl—on both basal planes and edges [73]. As shown in Figure 4b, these groups provide reactive sites for hydrogen-bond formation with NC. Hydroxyl moieties in NCs act as hydrogen donors, while oxygenated sites on GO serve as acceptors, forming dense interfacial hydrogen-bond networks (Figure 4c, red regions) [74,75,76]. These bonds promote intimate contact between components and enhance interfacial stability. Fourier-transform infrared spectroscopy provides direct evidence of these interactions. In self-assembled CNC–GO systems, the hydrogen-bond-associated absorption band shifts from approximately 1622 cm^−1^ (for CNC) and 1619 cm^−1^ (for GO) to around 1570 cm^−1^ in the composite, confirming the strengthening of interfacial hydrogen bonding [77].

The degree of interfacial bonding is strongly correlated with the surface charge of NC, suggesting that chemical functionalization can be used to further optimize interfacial compatibility. In addition to hydrogen bonding, van der Waals forces and hydrophobic/π–π stacking interactions also contribute significantly to interface stabilization. As illustrated in the structural model in Figure 4d, multiple types of noncovalent forces coexist between NC molecular chains and graphene layers, collectively maintaining the structural integrity of the composite. Yusuf et al. [78] demonstrated that the aromatic π-planes of GO engage in π–π stacking and hydrophobic interactions with the hydrophobic microdomains of cellulose, while van der Waals attractions reinforce interlayer cohesion. Xiong et al. [79] further reported that the synergistic effects of hydrogen bonding, van der Waals, and hydrophobic interactions are critical to uniform GO dispersion and long-term structural stability during membrane operation. Moreover, partially reduced GO retains sp^2^-hybridized carbon domains capable of weak hydrophobic interactions with cellulose surfaces [80]. The collective contribution of these forces results in a robust and stable interfacial network.

A well-engineered interface not only enhances the mechanical stability but also facilitates functional realization. Beyond direct NC–GO interactions, noncovalent modifiers such as sodium lignosulfonate, sodium carboxymethyl cellulose, and pyrene-functionalized hydroxypropyl cellulose can further tailor the surface properties, as illustrated in Figure 4e. These modifiers interact with both graphene and the cellulose matrix via specific functional groups, improving dispersion and interfacial compatibility. Owing to its one-dimensional morphology and high specific surface area, NC can partially adsorb onto or wrap around graphene sheets [81], physically preventing π–π-induced restacking and reducing aggregation tendencies. Consequently, NC serves not only as a reinforcing phase but also as an efficient nanodispersant, maintaining graphene sheets in uniformly distributed mono- or few-layer states. This architecture enhances the effective surface area and provides structural support for mass transport and anti-fouling performance [71]. Yifira et al. [82] confirmed that CNC coatings on reduced GO (rGO) markedly improved the dispersion stability, resulting in membranes with superior selectivity and anti-fouling resistance. Similarly, Zou et al. [83] fabricated CNC–GO membranes via in situ self-assembly, demonstrating uniformly distributed rGO sheets, stable pore structures, and enhanced long-term separation performance. Overall, interfacial networks dominated by hydrogen bonding and reinforced by van der Waals and π–π interactions constitute the molecular foundation for synergistic functionality in NC–GD composite systems.

### 4.2. Mass Transfer and Separation

Mass transfer and separation mechanisms lie at the core of the high performance of NC–GD membranes. These composites effectively overcome the classical permeability–selectivity trade-off commonly observed in polymeric membranes [86]. Their breakthrough originates from the formation of highly tunable nanoscale transport channels.

**Figure 5:**
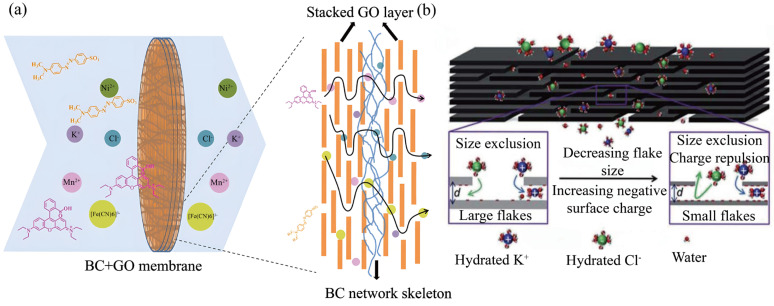
Mass transfer and separation mechanism diagram. (**a**) Schematic diagram of the permeation process of different organic or inorganic ions through BC + GO membrane; (**b**) comparison of size exclusion and ion charge repulsion effects between small-flake graphene oxide and large-flake graphene oxide during material permeation process. Image (**a**) reproduced with permission from Ref. [61]; image (**b**) reproduced with permission from Ref. [96].

Mass transfer primarily occurs within nanochannels located between stacked graphene sheets or co-constructed with the NC framework. The two-dimensional lamellar structure of GO forms the structural basis for size-selective sieving [87,88]. Ideally aligned GO layers generate sub-nanometer capillary channels that enable rapid water permeation while rejecting larger solutes such as hydrated ions and organic macromolecules. Figure 5a schematically illustrates the permeation process of different organic or inorganic ions through a BC+GO membrane, highlighting the size-based exclusion mechanisms. Nair et al. [89] first demonstrated that laminated GO membranes efficiently retained macromolecules and certain salts through molecular sieving. Later, Joshi et al. [90] correlated hydrated ion mobility with GO interlayer spacing, confirming the role of channel dimensions in ion selectivity. However, pure GO membranes tend to swell in aqueous environments, enlarging the interlayer spacing and thereby diminishing rejection of small solutes, especially monovalent ions, representing a major limitation in GO-based membranes. Incorporating NC effectively mitigates this issue. Hydroxyl and carboxyl groups on NC form hydrogen bonds or electrostatic interactions with GO layers, acting as “molecular rivets” that constrain interlayer movement. This prevents swelling and stabilizes the channel architecture. Gao et al. [67] reported that adding CNC to GO membranes reduced the interlayer expansion, increasing Na_2_SO_4_ rejection from 41% to 64%, thus confirming NC’s structural stabilization effect. Similarly, a ternary hydrogel membrane constructed from chitosan-immobilized graphene oxide and carboxymethyl cellulose achieved efficient recovery of Zn(II) ions from aqueous solution, owing to the reinforced channel structure formed through the synergistic interactions between NC and GO [91]. Additionally, the high hydrophilicity of NC introduces supplementary hydrophilic microchannels between GO layers, providing preferential pathways for water molecules and reducing mass transfer resistance [92]. In a comparable study, CTA–GO pervaporation desalination membranes exhibited enhanced permeation and desalination efficiency, with a reported water flux of 22.6 kg/m^2^·h and salt rejection of 98.1% at 60 °C, further highlighting the role of GO in facilitating high-efficiency water transport [93].

Surface charge characteristics further influence the separation performance; the enhanced ion charge repulsion effect resulting from a higher surface negative charge is further illustrated in Figure 5b. Both GO and NC carry negative charges derived from surface carboxyl and hydroxyl groups. This induces electrostatic repulsion toward negatively charged contaminants such as humic acid or anionic dyes, improving selectivity. Ren et al. [94] achieved 94.2% humic acid removal at 2 bar using a negatively charged GO membrane, attributing the performance to electrostatic repulsion. Similarly, adjusting the surface charge via polyelectrolyte coatings such as Poly(diallyldimethylammonium chloride) or Poly(sodium 4-styrenesulfonate) significantly modulated the ion permeability (e.g., Mg^2+^, Na^+^) [95].

Thus, NC–GD composite membranes integrate size exclusion, channel stabilization, and electrostatic repulsion to construct efficient water transport and selective separation systems. These cooperative mechanisms underlie their superior permeability, selectivity, and stability in water treatment applications.

### 4.3. Anti-Fouling and Antibacterial Properties

Biofouling remains one of the most persistent challenges to long-term membrane operation [97]. NC–GD composite membranes exhibit exceptional anti-fouling and antibacterial properties owing to their multifunctional interfaces, which act through both physical and chemical pathways.

The physical fouling resistance arises primarily from the enhanced surface hydrophilicity. Hydrophilic functional groups form a stable hydration layer that creates steric and energetic barriers against the adhesion of hydrophobic foulants such as proteins, bacteria, and oils. Both NC and GO contain abundant hydroxyl and carboxyl groups, and their combination substantially increases surface hydrophilicity. Feng et al. [98] embedded CNC into a PA-selective layer via interfacial polymerization, obtaining a thin-film nanocomposite-25 nanofiltration membrane with outstanding fouling resistance–flux recovery rates of 97.8%, 85.1%, and 91.5% for humic acid, bovine serum albumin, and sodium alginate, respectively. Liu et al. [99] fabricated a CNF–GO composite membrane with an ultrathin GO coating, significantly enhancing the mechanical stability, flux, and adsorption capacity.

**Figure 6:**
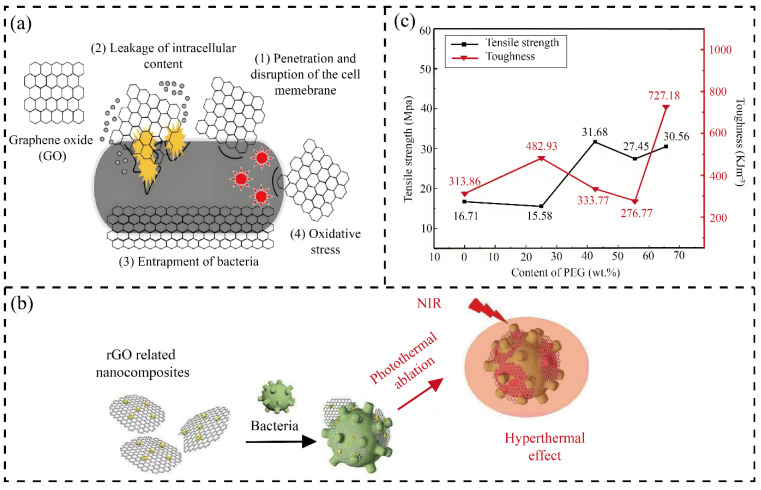
Antibacterial mechanisms and performance of NC–GD membranes. (**a**) GO antibacterial mechanisms: (1) membrane penetration/disruption, (2) intracellular content leakage, (3) ROS-induced oxidative stress, and (4) bacterial entrapment (wrapping effect); (**b**) rGO-based photothermal sterilization; (**c**) mechanical properties of rGO–CNC composite membrane. Image (**a**) reproduced with permission from Ref. [102]; image (**b**) reproduced with permission from Ref. [103]; image (**c**) reproduced with permission from Ref. [104].

Antibacterial functionality primarily originates from graphene derivatives through three synergistic mechanisms. First is physical disruption—the sharp edges of graphene nanosheets puncture bacterial membranes, causing cytoplasmic leakage [100]. Second is oxidative stress—GO and rGO generate reactive oxygen species that oxidatively damage proteins, lipids, and DNA, leading to bacterial death [101]. Third is photothermal inactivation—rGO’s high photothermal conversion enables localized heating under near-infrared irradiation, further enhancing the bactericidal efficacy [100]. These mechanisms are visually summarized in Figure 6a,b, which illustrate key processes such as membrane penetration, oxidative stress, and photothermal sterilization. Within NC–GD composites, the NC network prevents graphene sheet aggregation, ensuring uniform exposure of antibacterial sites and maintaining long-term effectiveness. Mir et al. [69] demonstrated complete inhibition of *E. coli* on BC–rGO membranes, whereas pure cellulose membranes supported bacterial growth, clearly confirming the dual physical–chemical mechanism. Therefore, NC–GD composites combine hydrophilic anti-fouling and graphene-based antibacterial functions to achieve durable biofouling resistance in complex aqueous environments.

### 4.4. Mechanical Stability

In pressurized separation processes, membranes must maintain sufficient mechanical strength and structural integrity to withstand operational stresses and repeated cleaning cycles. NC–GD composite membranes display superior mechanical performance compared to conventional polymeric membranes, primarily due to synergistic reinforcement between the NC and graphene components [105].

Mechanical enhancement stems from two key aspects: the intrinsic high modulus of the reinforcing phases and efficient interfacial stress transfer. Graphene possesses an ultrahigh Young’s modulus and fracture strength, while highly crystalline NC—particularly CNC—exhibits remarkable specific stiffness [106]. When integrated into membrane matrices, these nanofillers form interconnected load-bearing frameworks. However, their effectiveness depends on interfacial coupling. Hydrogen-bond networks between the hydroxyl groups of NC and the oxygenated groups of GDs facilitate stress transfer from the softer polymeric regions to the rigid nanofillers under external loading [107]. This mechanism suppresses microcrack propagation, enhancing the tensile strength, Young’s modulus, and fracture toughness.

As illustrated in Figure 6c, Xie et al. [104] fabricated a CNC-rGO–poly(ethylene glycol)-4 composite film exhibiting a tensile strength of 30.56 MPa at only 21 μm thickness—an 83% improvement over pure poly(ethylene glycol) films. The hierarchical network formed by one-dimensional NC and two-dimensional graphene nanosheets establishes a multiscale reinforcement architecture, conferring exceptional mechanical stability and long-term durability, even under harsh operating conditions.

### 4.5. Synergistic Coupling of Functional Mechanisms

The four major functional mechanisms—interfacial interaction, mass transfer, anti-fouling/antibacterial performance, and mechanical reinforcement—are not isolated phenomena but are tightly interlinked through interfacial structural coupling.

The hydrogen-bonded interfacial network acts as the nexus of this synergy. Dense hydrogen bonds between NC and GDs restrict GO swelling, stabilize sub-nanometer channels, and maintain mass-transfer pathways, while simultaneously facilitating stress transfer across the matrix. This dual function ensures both channel stability and mechanical reinforcement. Meanwhile, mass-transfer and anti-fouling mechanisms act cooperatively: negatively charged carboxyl and hydroxyl groups on NC and GO surfaces induce electrostatic repulsion and sustain hydration layers that deter foulant adhesion, enabling high flux and flux recovery. Similarly, antibacterial and mechanical mechanisms are interconnected. Interfacial hydrogen bonding stabilizes GO edges, preserving antibacterial sharpness while preventing detachment. The hydrophilic NC network distributes antimicrobial regions uniformly, maintaining both activity and structural robustness.

In essence, the superior permeability, selectivity, and durability of NC–GD composite membranes originate from these multi-mechanism synergies. Interfacial chemistry governs structural integrity; coupled mass-transfer and fouling-resistance mechanisms enhance separation performance; and the combination of mechanical and antibacterial stability ensures long-term operational reliability. Together, these coupled effects establish NC–GD composites as an advanced platform for next-generation, high-performance, and sustainable membrane technologies.

## 5. Typical Water Treatment Applications

### 5.1. Pollutant Adsorption

The high adsorption efficiency of NC–GD composite membranes arises from their abundant surface functionalities and strong interfacial interactions. Both NC and GO layers contain numerous hydroxyl and carboxyl groups, which provide active sites for capturing heavy metal ions such as Cd^2+^ and Pb^2+^ through electrostatic attraction or complexation. For organic dyes and antibiotics, the large specific surface area of GO offers ample adsorption sites, while hydrogen bonding and π–π interactions between NC and GO further enhance molecular affinity. This synergistic mechanism enables the simultaneous capture of various pollutants, while the membrane configuration facilitates easy solid–liquid separation and material regeneration.

GO–BC composite membranes, for instance, exhibit remarkable adsorption capacities for organic dyes such as Rhodamine B and Methyl Orange due to their three-dimensional porous structure and rich oxygen functionalities. These membranes maintain structural integrity and recyclability, making them ideal models for dye removal [108]. Similarly, CNC–GO hybrids have demonstrated >80% removal efficiency for antibiotics under optimized pH conditions, with well-defined adsorption isotherms and kinetics supporting the quantitative evaluation of adsorption behavior [109]. Recent studies show that NC–GD composites effectively remove trace organic contaminants. Crystalline nanocellulose anchored on rGO efficiently adsorbs pharmaceutical micropollutants (e.g., aspirin, acetaminophen), combining high adsorption capacity with mechanical stability for regeneration. These results build on earlier CNC–GO antibiotic removal studies, pointing to scalable adsorbent applications [110].

For inorganic pollutants, GO–cellulose membranes efficiently remove divalent metal ions (Cd^2+^, Pb^2+^, Cu^2+^) via chelation and electrostatic interactions involving carboxyl and hydroxyl sites on both components [111]. Introducing polyethylenimine onto TEMPO-oxidized CN and GO frameworks further enhances adsorption by providing positively charged sites. These self-assembled hydrogels effectively remove both dyes and heavy metals, achieving synergistic multi-pollutant purification [112], the adsorption of different pollutants is shown in Table 3. Collectively, these studies validate the versatility and high efficiency of NC–GO composites in removing dyes, organic contaminants, and heavy metals, while highlighting tunable adsorption performance through interfacial functionalization and structural design.

### 5.2. Smart Oil–Water Separation

NC–GD composite membranes also exhibit exceptional performance in smart oil–water separation, enabled by the coupling of surface wettability control and nanoscale sieving. Abundant hydrophilic groups on NC and GO surfaces endow the composite with superhydrophilicity, forming a hydration layer that effectively repels hydrophobic oil droplets. During separation, this surface chemistry synergizes with the hierarchical porous channels of the NC network and the lamellar GO structure, allowing water molecules to pass rapidly while blocking oil phases. Recent studies show that nanocellulose aerogels and graphene nanomesh membranes excel in oil–water separation, with high efficiency and cycle stability. These results suggest potential for reusable sorbents and flow-compatible membranes in produced water and spill remediation [113,114].

Mir et al. [69] reported that BC–GO composite membranes achieve >97% oil–water separation efficiency, maintaining performance over 10 cycles due to the robust hydrogen-bonded interface between GO and the BC matrix. Dawwam et al. [70] demonstrated near-instantaneous separation—within 5 s—of n-heptane/water mixtures, attributing this to the hydrophilic–superoleophobic character induced by GO. Such rapid efficient separation illustrates the potential of these composites for intelligent and scalable oil–water separation systems.

Furthermore, CNF–GO-based superhydrophobic aerogels prepared by Qiao et al. [115] achieved >98% separation efficiency and retained >90% efficiency after 15 reuse cycles. The integration of hierarchical porosity with surface chemistry resolved the classical trade-off between flux and selectivity, providing an effective structural model for next-generation separation membranes.

### 5.3. Antibacterial and Anti-Fouling Membranes

The antibacterial and anti-fouling properties of NC–GD membranes originate from synergistic surface mechanisms coupled with superior mechanical stability. Enhanced surface hydrophilicity creates a hydration barrier that resists foulant deposition, while GO contributes chemical and physical antibacterial activity through direct cell membrane disruption and oxidative stress. The interfacial hydrogen-bonding network between NC and GO ensures uniform GO dispersion and strengthens the wet mechanical stability.

Yusuf et al. [78] reviewed the design of NC–GO composites, highlighting that surface amination, metal nanoparticle (Ag, Cu) incorporation, and cationic polymer grafting substantially improve antibacterial efficacy. GO–metal nanocomposites provide dual mechanisms—physical damage and ionic toxicity—suppressing bacterial adhesion and biofilm formation. For instance, Prakash et al. developed electro spun cellulose acetate nanofibers incorporating GO/TiO_2_/curcumin, which demonstrated excellent antibacterial and wound healing properties, further illustrating the potential of GO-based composites in biomedical and antibacterial applications [116]. Specifically, GO’s sharp edges physically rupture cell membranes, while its oxygen groups induce reactive oxygen species generation, leading to cellular damage. The addition of NC enhances hydrophilicity and maintains high permeability, reducing fouling while preserving antimicrobial functionality.

Mir et al. [69] showed that self-supporting BC–GO membranes exhibited <10% flux decline after 12 h of operation at 0.2 MPa, outperforming pristine BC membranes. The incorporation of GO reduced surface contact angles from 65° to 48°, improving the hydrophilicity and reducing organic and bacterial adhesion by >70%. Luz et al. [117] further demonstrated that GO–Ag membranes produced inhibition zones of 18–21 mm against *S. aureus* and *E. coli*, markedly larger than unmodified membranes (<5 mm). Similarly, Bhadane [118] reported >90% inhibition of Gram-negative bacteria with GO–cellulose acetate membranes, which maintained >85% flux recovery after five filtration cycles. Sheng et al. [119] developed a TEMPO-oxidized BC–GO nanochannel membrane that achieved <8% flux decay and efficient microbial removal, owing to enhanced carboxyl–GO interfacial bonding. Importantly, several recent works have progressed from static inhibition assays to dynamic filtration or long-term operation tests that quantify biofouling performance under realistic hydrodynamic stresses. For instance, long-term operation studies of GO-containing membranes demonstrated sustained purification performance over multi-week runs with controlled flux decline, providing stronger evidence that NC–GO hybrids can contribute to operationally relevant antifouling strategies [120,121].

Collectively, these findings confirm that the anti-fouling and antibacterial capabilities of NC–GO membranes arise from multiple coupled effects—GO’s physical and oxidative bactericidal actions and synergistic metal ion toxicity and NC’s hydrophilic and structural reinforcement. These mechanisms substantially prolong the flux stability and operational lifespan, positioning NC–GD composites as promising materials for advanced water treatment and biofouling mitigation.

### 5.4. Water Desalination

The controlled assembly of sub-nanometer channels with enhanced interfacial energy barriers is a key approach for low-energy seawater desalination. Porous reduced rGO layers reinforced by a CNF network were vacuum-assisted self-assembled to 0.74 nm interlayer spacing, intermediate between hydrated Na^+^ (0.72 nm) and free water (0.28 nm), achieving 98.3% NaCl rejection at 2 bar with 18.4 L m^−2^ h^−1^ flux [68]. CNF entanglement suppressed rGO π–π restacking and, via interfacial hydrogen bonding, limited flux decline to <2% over 120 h in 30 g L^−1^ NaCl, mitigating swelling-induced expansion of pure rGO membranes.

Janus structures were prepared by in situ reduction of rGO on electrospun CNFs. A porous rGO top layer (≈94% light absorption) converted solar energy to heat, while the hydrophilic CNF network maintained water transport. Gradient channels (50–200 μm) shortened vapor paths to <300 μm, achieving 3.28 kg m^−2^ h^−1^ evaporation under 1 kW m^−2^ irradiation with 91.5% energy efficiency [122]. The negatively charged CNF surface (ζ ≈ −42 mV) induced the Donnan exclusion of Na^+^, reducing the condensate salinity from 35,000 ppm to <0.5 ppm. Seven-day tests showed no surface crystallization, reflecting effective mechanical detachment and redissolution of salt nuclei.

CNCs (0.8 wt %) intercalated into GO membranes addressed antibiotic-induced horizontal gene transfer. CNC–COOH/GO–OH hydrogen bonding compressed interlayer spacing to 0.65 nm, below tetracycline (1.22 nm) and sulfamethoxazole (0.96 nm), achieving >95% antibiotic rejection [67]. The additional negative surface charge (−0.38 e nm^−2^) enhanced Mg^2+^/Ca^2+^ rejection to 99.1% while retaining ~60% Na^+^ permeability. In simulated seawater (35 g L^−1^ NaCl, 10 mg L^−1^ antibiotic), the flux remained 92% over 72 h with no CNC loss, demonstrating stability under high pressure (15 bar) and chlorine cleaning.

Collectively, size-selective sieving, charge exclusion, and photothermal synergy enable low-pressure (≤2 bar) or solar-driven desalination, suppress salt crystallization, and remove microcontaminants, providing a scalable platform for sustainable seawater treatment.

### 5.5. Emerging Separation Technology Applications

Beyond the typical water treatment scenarios, NC–GD composite membranes have shown promising potential in emerging separation technologies driven by their tunable nanochannels, synergistic interfacial properties, and multi-functional integration.

In osmotic energy conversion, the ordered nanochannels and charged surfaces of NC–GD membranes enable selective ion transport, forming concentration gradients for energy harvesting. Sheng et al. [119] fabricated TEMPO-oxidized BC–GO nanofiber membranes that achieved efficient ion sieving and osmotic power generation, with a power density of ~0.8 W m^−2^ under simulated salinity gradients, highlighting their applicability in blue energy exploitation. In gas separation, the compact layered structure of NC–GD composites provides molecular sieving effects for small gas molecules. Wang et al. [81] developed GO–nanocellulose–cellulose acetate mixed matrix membranes that exhibited enhanced CO_2_/N_2_ separation performance (CO_2_ permeability: 326 Barrer, CO_2_/N_2_ selectivity: 38), attributed to the synergistic regulation of the pore size by NC and GO and improved interfacial compatibility between the filler and matrix. These emerging applications lay the foundation for integration into multi-functional separation systems.

## 6. Challenges

### 6.1. Challenge I: Controlling GO Interlayer Swelling

Excessive hydration-induced swelling of GO layers remains a principal cause of selectivity loss. Although crosslinking agents (e.g., ethylenediamine) effectively anchor layers, they often block nanochannels, resulting in substantial flux reduction [123]. In contrast, NC intercalation maintains hydrophilicity and permeability but relies primarily on non-covalent bonding, limiting the long-term stability and small-ion rejection [124]. Achieving precise interlayer spacing control without compromising flux thus remains an unresolved challenge.

### 6.2. Challenge II: The “Double-Edged Sword” of NC Content

The reinforcing and dispersing effects of NCs depend strongly on the dosage, exhibiting nonlinear correlations with performance. At low loadings, NCs enhance mechanical strength and stabilize GO layers; excessive addition, however, increases porosity and disrupts compactness, sharply reducing the selectivity. Given the variability in NC morphology and preparation methods, establishing a universal quantitative model to determine optimal NC content is urgently required.

### 6.3. Challenge III: Cost–Performance Trade-Off in Scale-Up

Industrial application faces economic and environmental barriers. Conventional GO synthesis (e.g., Hummers’ method) yields high-quality materials but involves costly and hazardous reagents. Although green routes—such as enzymatic NC hydrolysis and electrochemical GO exfoliation—reduce the environmental impact, they often compromise performance (e.g., lower flux or weaker mechanical stability. Balancing economic feasibility, environmental sustainability, and functional excellence remains a central challenge for commercialization.

## 7. Conclusions

NC-GD composite membranes have demonstrated exceptional performance in water treatment, benefiting from the synergistic properties of nanocellulose and graphene derivatives. These membranes exhibit enhanced permeability, selective separation, mechanical stability, and fouling resistance, owing to the optimized interfacial chemistry and structural integrity achieved through various fabrication methods. Despite significant progress, challenges remain, particularly in controlling graphene oxide interlayer swelling, optimizing NC content for large-scale production, and balancing performance with cost-effectiveness. Future research should focus on adaptive interfacial design, scalable green fabrication techniques, and the precise coupling of multifunctional mechanisms to unlock the full potential of NC-GD composites for sustainable high-performance water purification applications.

## Figures and Tables

**Table 1:** NC–GD film preparation methods: a comparison.

Method	Suitable NC–GD Combination	Pros	Cons
Vacuum filtration [52]	CNC-GO/rGO (Rigid Scaffold)	1. Simple operation 2. Controllable structure 3. Forms dense ordered layered structures 4. High separation selectivity	1. Film thickness control depends on parameters; flexible materials are prone to agglomeration 2. Batch processing is challenging for continuous production
Layer-by-layer self-assembly [53]	CNF-GO (Surface Charge Tunable)	1. Precise control over film thickness and morphology 2. Enables easy fabrication of ultrathin films 3. Structurally robust	1. Time-consuming process is unsuitable for mass production 2. Requires prior design and regulation of surface charge
Solution casting [54]	CNF/BC and rGO/aminated GO (Well-functionalized)	1. Intuitive and straightforward process 2. Facilitates formation of structurally uniform films	1. Poor control over film thickness and pore structure 2. Challenges exist for large-scale production

**Table 2:** Comparative analysis of core performance for different NC–GD composite membranes.

NC–GD Combination	Water Flux	Separation Performance	Adsorption Capacity	Stability	Ref.
CNC–GO	Permeability increased by 2 to 4 times	Antibiotic rejection: >95%: divalent ion (Mg^2+^/Ca^2+^) rejection: 99.1%	—	—	[67]
CNC–rGO/PEO	18.4 L m^−2^ h^−1^ @ 2 bar	NaCl removal rate: 98.3%	—	The flux decrease within 120 h is less than 2%.	[68]
CNF–GO	Five times higher than pure CNF membrane	For positive/negative dyes >90%	—	—	[64]
TOCNF–nanoGO	The filtration rate is 111 times that of the GO composite membrane	Suitable for dye/macromolecule separation	Cu(II): 68.1 mg/g	Stable; structure retained after three cycles.	[56]
BC	0.70 ± 0.27 L m^−2^ h^−1^ kPa^−1^	BSA protein: 98 ± 2%	—	Stable; flux recovery rate after three cycles: 68 ± 16%.	[9]
BC–rGO	394.6 L·m^−2^·h^−1^ @ 2 bar	Organic matter and bacterial removal rate >95%	—	Antibacterial; flux recovery rate after five cycles >95%.	[69]
BC–GO	—	Oil separation: 90% (within 5 s)	—	—	[70]

**Table 3:** Pollutant adsorption summary.

Pollutant Type	Water Source	NC–GD Combination	Removal Efficiency	Ref.
Rhodamine B	Textile dyeing wastewater	BC–GO	99.50% at pH 3	[108]
Levofloxacin hydrochloride (antibiotic)	Antibiotic-containing wastewater	CNCs–GO	>80.1% At optimal conditions (pH 4, dosage 1.0 g L^−1^, 4 h)	[109]
Divalent metal ions (Co^2+^, Ni^2+^, Cu^2+^, Zn^2+^, Cd^2+^, Pb^2+^)	Advanced purification of drinking water	GO–cellulose	Maximum adsorption capacities: Co^2+^: 15.5 mg·g^−1^ Ni^2+^: 14.3 mg·g^−1^ Cu^2+^: 26.6 mg·g^−1^ Zn^2+^: 16.7 mg·g^−1^ Cd^2+^: 26.8 mg·g^−1^ Pb^2+^: 107.9 mg·g^−1^ at pH 4.5	[111]

## Data Availability

No new data were created or analyzed in this study.

## Abbreviations

NC	Nanocellulose
GDs	Graphene derivatives
CNC	Cellulose nanocrystals
CNF	Cellulose nanofibrils
BC	Bacterial cellulose
GO	Graphene oxide
rGO	Reduced graphene oxide
TEMPO	2,2,6,6-tetramethylpiperidin-1-yl-oxyl
TOCNF	TEMPO-oxidized CNF
PVDF	Polyvinylidene fluoride
PES	Polyether sulfone
PA	Polyamide
RO	Reverse osmosis
LbL	Layer-by-layer
